# Sharing of Verified Information about COVID-19 on Social Network Sites: A Social Exchange Theory Perspective

**DOI:** 10.3390/ijerph18031260

**Published:** 2021-01-31

**Authors:** Jiabei Xia, Tailai Wu, Liqin Zhou

**Affiliations:** School of Medicine and Health Management, Tongji Medical College, Huazhong University of Science and Technology, Wuhan 430000, China or bei_xx@sina.com (J.X.); wutailai@hust.edu.cn (T.W.)

**Keywords:** COVID-19, verified information sharing, social exchange theory, social networking sites

## Abstract

*Background*: Verified and authentic information about coronavirus disease (COVID-19) on social networking sites (SNS) could help people make appropriate decisions to protect themselves. However, little is known about what factors influence people’s sharing of verified information about COVID-19. Thus, the purpose of this study was to explore the factors that influence people’s sharing of verified information about COVID-19 on social networking sites. *Methods*: Based on social exchange theory, we explore the factors that influence sharing of verified information about COVID-19 from two perspectives: benefits and costs. We employed the survey method to validate our hypothesized relationships. By using our developed measurement instruments, we collected 347 valid responses from SNS users and utilized the partial least squares method to analyze the data. *Results*: Among the benefits of sharing verified information about COVID-19, enjoyment in helping (β = 0.357, *p* = 0.000), altruism (β = 0.133, *p* = 0.029) and reputation (β = 0.202, *p* = 0.000) were significantly associated with verified information sharing about COVID-19. Regarding the costs of sharing verified information about COVID-19, both verification cost (β = −0.078, *p* = 0.046) and executional cost (β = −0.126, *p* = 0.011) also significantly affect verified information sharing about COVID-19. All the proposed hypotheses were supported. *Conclusions*: By exploring factors from both benefits and costs perspectives, we could understand users’ intention to share verified information about COVID-19 comprehensively. This study not only contributes to the literature on information sharing, but also has implications concerning users’ behaviors on SNS.

## 1. Introduction

Since December 2019, the coronavirus pandemic (COVID-19) has caused high morbidity and mortality in more than 30 countries around the world [1]. The COVID-19 pandemic has been widespread and devastating, seriously affecting people’s daily life. Social networking sites (SNS) have played an important role in major crisis events [2]. People use SNS to seek information about the crisis, discuss and share personal experiences, and interact with other users regarding issues related to the crisis [2]. Therefore, as an important communication channel, people exchange information about COVID-19 on their SNS.

However, the diffused information about COVID-19 on SNS contains a lot of disinformation and misinformation [3], thus creating an information disaster. According to reports, more than 1 million Internet users are committed to spreading rumors and unconfirmed information about COVID-19 [4]. The spread of this misinformation or disinformation will not only create panic for the public [5], but also degrade the information environment on the SNS [3]. Therefore, it is important to share verified information about COVID-19 on SNS. For the public, sharing verified information about COVID-19 would not only provide profound knowledge value, but also improve the public’s ability to fight COVID-19. For the social network environment, reducing the dissemination of disinformation and misinformation on SNS could effectively purify the information environment.

Previous literature has considered the sharing of information about COVID-19. Sharing misinformation [6] and disinformation [7] about COVID-19, not only exacerbates cyberchondria [6], but also brings chaos to society [8]. Therefore, it is necessary to reduce the dissemination of misinformation and disinformation about COVID-19 and encourage the sharing of verified COVID-19 information. However, little of the previous literature considers sharing verified COVID-19 information. Relevant literature about information and knowledge sharing in other contexts has also explored the factors impacting people’s sharing behavior. Factors influencing information sharing in other contexts include trust [9], ease of use [10], information quality and psychological contract [11], while factors like community identification [12], organizational commitment [13], sharing self-efficacy [14], cognitive cost [15], actual cost [16] are revealed to influence knowledge sharing. However, sharing verified of information about COVID-19 in SNS is still not discussed well in the information and knowledge sharing literature. Combining this fact with the importance of sharing verified COVID-19 information, we propose the following research question:

*What are the factors influencing the sharing verified information on COVID-19 in SNS?*

To address the research question, we first conducted a literature review about our study and discuss social exchange theory as our theoretical perspective. Then, we establish our research model by developing the corresponding hypotheses. Analysis methods and results follow. A discussion and the conclusions of this study are given the last sections.

## 2. Theoretical Foundation

### 2.1. Literature Review

The literature related to our study could be classified into three streams: sharing of information about COVID-19 on SNS, information sharing in other contexts and knowledge sharing. First, we address the literature about sharing COVID-19 information. The previous literature shows that disinformation and misinformation sharing about COVID-19 exists on SNS [3], which can cause panic and anxiety for the public [17]. Several factors of sharing COVID-19 information are explored. For example, Islam et al. found that entertainment and self-promotion could promote the sharing of misinformation about COVID-19 [18]. Apuke and Omar found that altruism, instant news sharing, socialisation predicted fake news sharing related to the COVID-19 pandemic among social media users in Nigeria [19]. Laato et al. suggested that a person’s trust in online information and perceived information overload were strong predictors of unverified information sharing [6]. Therefore, the sharing of verified information about COVID-19 is not well studied and needs further exploration.

Second, literature about information sharing in other contexts is reflected in many research fields, such as supply chain management [20], network investment [21] and social media [22]. For example, Oh and Syn found that enjoyment, self-efficacy, altruism, community interest, reciprocity and reputation could encourage users’ information sharing [23]. Lin et al. showed that social media design features, security and personal factors were the determinants that affect users’ attitude and information sharing behavior [24]. Thus, literature in this stream has little so say about the sharing of verified COVID-19 information.

Third, the literature about knowledge sharing mainly takes the social exchange theory as its theoretical foundation, and discusses the factors that drive knowledge sharing behavior in a virtual environment [15,25,26]. For example, Yan et al. proposed that members in online health communities shared knowledge from the perspective of benefits and costs [15]. Kankanhalli et al. suggested that factors from benefit and cost perspectives significantly impacted EKR usage by knowledge contributors [26]. Summarizing the above literature, there is a gap about sharing verified COVID-19 information on SNS. We provide a quite comprehensive review of the relevant literatures in Appendix A.

### 2.2. Social Exchange Theory

Social exchange theory (SET) provides the theoretical basis for this study. Social exchange theory was developed from exchange theory [27]. From an economic point of view, exchange theory emphasizes that people evaluate the potential costs and benefits of an exchange to obtain the best benefits [27]. However, unlike economic exchanges, in a social exchange, people do not always expect tangible returns from the social interaction, but prefer to exchange their expertise or effort for intangible returns, such as status and respect [27]. At the same time, social exchanges cannot guarantee that the return is equal to the cost, but rather is based on the belief of mutual return [28].

In this study, sharing verified information on COVID-19 could be treated as a social exchange since people sharing verified information on COVID-19 would gain some social benefits with costs on SNS. Therefore, we could utilize social exchange theory as the theoretical foundation to understand the sharing of verified information about COVID-19. Meanwhile, social exchange theory is validated in other contexts, including supply chain management [29], consumer behavior [30], knowledge sharing [31] and online communities [32]. Therefore, it would be proper to make use of social exchange theory in our study. Based on social exchange theory, we explore the factors influencing the sharing of verified COVID-19 information from the benefit and cost perspectives and construct our research model in the following section.

## 3. Research Model and Hypotheses Development

**Figure 1:**
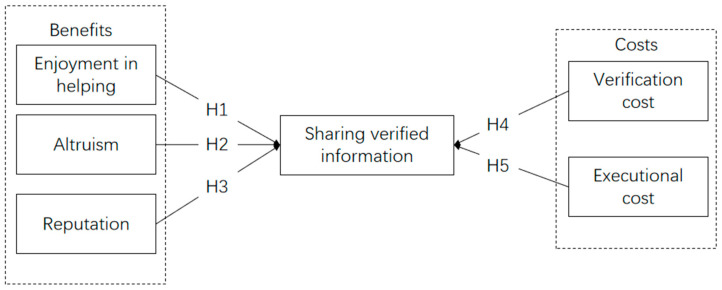
Research model and hypothesized relationships.

Based on social exchange theory, we propose a research framework for the sharing of verified information about COVID-19 from the perspective of benefits and costs. For the benefit perspective, we hypothesize the enjoyment in helping, altruism and reputation influence from sharing verified COVID-19 information. From the cost perspective, verification cost and executional cost are assumed to influence the sharing of verified information about COVID-19. The hypothetical relationship is shown in Figure 1.

### 3.1. Benefits of Sharing Verified Information

#### 3.1.1. Enjoyment in Helping

Enjoyment in helping refers to an inner enjoyment of helping others without asking for anything in return [26]. Enjoyment is a self-motivation and internal factor that makes people feel happy and enthusiastic even without external or tangible compensation when engaging in certain behaviors [23]. People who enjoy helping others are more willing to share verified COVID-19 information rather than gaining other external rewards on SNS. Therefore, enjoyment in helping others could be considered an intrinsic motivation to share information in social networks [33]. Therefore, we can hypothesize the following:

**Hypothesis 1** **(H1).** *Enjoyment in helping positively influences the sharing of verified information about COVID-19 on SNS.*

#### 3.1.2. Altruism

Altruism refers to voluntary actions to help others without expecting anything in return [25]. People with altruistic ideals will spontaneously share verified information about COVID-19 and see it as a way to help others without requiring any reward. Prior study has shown that altruistic individuals promote knowledge sharing within an organization [26]. Altruism is the most influential motive for knowledge sharing on virtual networks [34]. Therefore, altruism is conducive to enhancing sharing of verified COVID-19 information. As a result, we can hypothesize that:

**Hypothesis 2** **(H2).** *Altruism positively influences the sharing of verified information about COVID-19 on SNS.*

#### 3.1.3. Reputation

Reputation is an external reward that motivates people to contribute and share knowledge on social networks [23]. Studies have shown that in virtual communities, gaining status, reputation and respect are important driving factors for many users to participate in knowledge-sharing activities [35]. When users find that sharing verified information about COVID-19 could help them gain recognition from their peers on SNS, they would perceive a benefit from the recognition. Such perceived benefit may drive them to share verified information on COVID-19 in SNS. Thus, we hypothesize that:

**Hypothesis 3** **(H3).** *Reputation positively influences the sharing of verified information about COVID-19 on SNS.*

### 3.2. Cost on Sharing Verified Information

#### 3.2.1. Verification Cost

Verification cost can be assessed from the expertise, time, and energy required to verify the information about COVID-19. When users spend time, energy and expertise to verify the authenticity of information about COVID-19, they would feel a loss in their mind which may result in a decrease in the willingness to share verified COVID-19 information on. Therefore, we hypothesize that:

**Hypothesis 4** **(H4).** *Verification cost negatively influences the sharing of verified information about COVID-19 on SNS.*

#### 3.2.2. Executional Cost

Executional costs include the time, material, and financial resources that individuals commit when they engage in certain activities [15]. After users verify or gain verified information about COVID-19, they would execute the sharing behavior on the SNS. To execute the sharing behavior, users would spend their time, energy or resources. Previous studies have shown that when the knowledge contribution requires significant time, sharing tends to be inhibited [36]. The cost would make them feel a loss. As a result, there will be little willingness to share verified information about COVID-19. Consequently, we hypothesize that:

**Hypothesis 5** **(H5).** *Executional cost negatively influences the sharing of verified information about COVID-19 on SNS.*

## 4. Methods

### 4.1. Measurement Instrument

The investigation method was adopted in this study. The survey instrument was developed by adapting some previously validated scales to our research context. Items for enjoyment in helping were adapted from Kankanhalli et al. [26], items for altruism were from Chang and Chuang [25]. Items for reputation were from Zhang et al. [37]. Items for verification cost were adapted from Sun et al. [16]. Items for executional cost were from Yan et al. [15]. Finally, items for sharing of verified information were from Lin and Wang [38]. All items were measured by using a 5-points Likert scale with values ranging from “1 = strongly disagree” to “5 = strongly agree”.

Since the survey instrument was originally developed in English and we plan to collect data in China, we use the backtranslation method to translate it into Chinese [39]. The English instrument is translated into Chinese for the first time by a bilingual author. Then, another bilingual author back-translated the Chinese version into English. The two authors then compared the two English versions to check for inconsistencies and resolve them through discussion. After confirming the translated survey instrument, a pretest was conducted by interviewing three experts in medical informatics and information systems. Seventeen social network site users were also surveyed in the pretest. We revised the questionnaire according to their comments and suggestions. The survey instrument is presented in Supplementary Table S1.

### 4.2. Data Collection

Given China was the first country to suffer from COVID-19 and has the largest number of internet users in the world, we decided to collect data for this study from China [40]. To access users of social network sites efficiently, we employed a paid survey service from a leading online market research company. Market research companies can effectively manage online surveys and recruit voluntary, active and diverse research participants for different research purposes [40]. Since the purpose of this study was to investigate users’ verified information about COVID-19 in social networking sites, we randomly invited respondents with experience in sharing verified information about COVID-19 on social network sites to fill out our questionnaire. The study procedure was approved by the Institutional Review Committee of Tongji Medical College, Huazhong University of Science and Technology (No. 2017S319). During the three weeks of deployment, we received a total of 381 responses.

To ensure the quality of data collection, several actions are taken during the data collection. First, attention-traps and reverse-coded questions were used in the questionnaire to check whether respondents were reading all the questions completely and responding honestly. Second, several screening questions were set to check whether the respondents shared information about COVID-19 such as “whether you have shared information about COVID-19 on social network sites”, “which social network sites do you use most”. Finally, the cases with missing values or similar values for all questions were not included. Thus, we are left with 347 complete and valid responses. The demographic information of our final sample is summarized in Table 1.

## 5. Results

### 5.1. Reliability and Validity

This study uses the partial least square (PLS) technique to analyze the data. We conducted the confirmatory factor analysis by using SmartPLS 3.3.0 [41]. The results of reliability and convergent validity are given in Table 2. The values of Cronbach’s alpha and composite reliabilities are all above 0.7, thus, confirming the good reliability for the model [42]. Meanwhile, the values of average variance extracted (AVE) of each structure are all above 0.5, and loadings for each item are also all above 0.7, thus, reflecting good convergence validity [43]. Furthermore, Table 3 presents the analysis results on discriminant validity. The square root of the AVE values of each latent variable in the model are larger than the correlation coefficient, which indicates that the measurement model has a good discriminant validity [44]. Hence, we conclude that the quality of measurement model is adequate for testing hypothesized relationships.

To examine whether common method bias is an issue in our study. First, we conducted Harman’s single-factor test using principle component analysis in SPSS 18.0 (company, city, state abbrev if USA, country). The analysis showed five factors were extracted. The first factor in the unrotated solution explained 31.2% of the variance, which is less than 50% [45]. Second, a marker variable technique was also used to test the common method bias. We selected organizational commitment as the marker variable which is not related to our research model. The analysis result showed that organizational commitment did not associated with sharing verified information on COVID-19 significantly. Therefore, common method bias was not a problem in our study.

### 5.2. Structural Model Analysis

**Figure 2:**
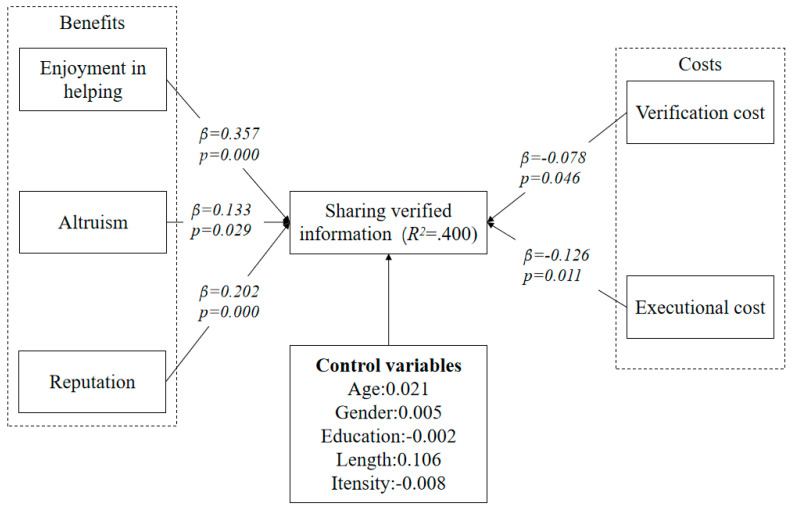
Analysis results of the hypothesized model.

By conducting the bootstrapping analysis in PLS, we test the hypothesized relationships in this study (Table 4). The analysis results are shown in Figure 2. From the perspective of benefits, enjoyment in helping, altruism and reputation were all found to influence sharing verified information significantly. Therefore, H1, H2 and H3 are supported. Meanwhile, regarding the perspective of cost, the analysis results show that both verification cost and executional cost significantly affect verified information sharing. Therefore, H4 and H5 are supported. Therefore, the analysis results convey that social exchange theory is validated in our study. Meanwhile, we also consider the effect of some control variables including age, gender, education, length and intensity of using social networking sites and only find that length of social networking site use has significant effect on the sharing of verified information.

## 6. Discussion

This study explores the factors that influence users’ sharing of verified information about COVID-19 on social network sites. On the basis of social exchange theory, we propose the factors of sharing verified information in terms of benefits and costs. From the perspective of benefits, we propose enjoyment in helping (β = 0.357, *p* = 0.000), altruism (β = 0.133, *p* = 0.029) and reputation (β = 0.202, *p* = 0.000) directly influence users’ sharing of verified COVID-19 information on social networking sites. From the perspective of costs, we propose that verification cost (β = −0.078, *p* = 0.046) and executional cost (β = −0.126, *p* = 0.011) directly influence users’ sharing of verified information in social network sites. By using a survey method, we find that all the hypothesized relationships are manifested. These results imply that our research model can help adequately understand the factors of users’ sharing of verified information about COVID-19 in social network sites.

### 6.1. Implications

This study has both theoretical and practical implications. For the theoretical implications, this research makes several contributions to the literatures.

First, our study firstly explores the sharing of verified information about COVID-19 on social networking sites. Previous literature was more focused on the sharing of misinformation or disinformation or rumors about COVID-19 which might cause panic and anxiety for the public or information sharing in the supply chain management, network investment and social media contexts, but less about sharing of verified information about COVID-19. Given that sharing of verified COVID-19 information is important for fighting the consequences of COVID-19, our study fills an important research gap.

Second, we extend social exchange theory to study the sharing of verified information about COVID-19. Through a survey method, we validate our research model based on social exchange theory. The previous literature mainly applied social exchange theory in contexts like knowledge sharing, online community and consumer behaviors, but not to the sharing of unverified information about COVID-19 on SNS. Since all the hypothesized relationships are supported, social exchange theory is validated in the sharing of verified information context.

Finally, several factors driving the sharing of verified information about COVID-19 from the benefits and costs perspectives are explored. Enjoyment of helping, altruism and reputation are found to be beneficial factors that facilitate the sharing of verified information, while verification and executional costs are the main costs related to the sharing of verified information. The explored factors could provide direct answers to our research question.

Besides the theoretical implications, this study has practical utility. First, several actions could be taken to encourage the sharing of verified COVID-19 information based on the identified factors from a benefit perspective. For example, SNS could use gamification elements in their design to increase the enjoyment of sharing verified information, provide stamps or seals to recognize the verified information sharing behavior and provide bonus points to users who share verified information about COVID-19.

Second, SNS could try to reduce the costs of sharing verified information about COVID-19. For example, SNS could play the role of “guardian” and guide public opinion by creating some mechanisms to refute rumors about COVID-19. Meanwhile, SNS could create a convenient and fast sharing environment, improve the interface design and functional modules in the platform to make the information sharing process friendly and smooth.

Third, social network sites should combat the spread of unverified information about COVID-19 and clean up the information environment of social network sites. With the help of advanced information technology and management mechanisms, social network platforms can comprehensively utilize algorithms, AI and other technologies to reduce the release of unverified information. For example, companies such as Facebook and Google are working as developers to explore ways to mitigate the impact of fake news [46], which can help combat the spread of unverified information about COVID-19 and may reduce its impact on society and individuals.

### 6.2. Limitations and Future Directions

The limitations in this study can be the basis for future research directions. First, our study used a cross-sectional survey; we did not take the dynamics of the studied factors into consideration. Hence, future studies could use longitudinal research designs to identify the relationships between verified information sharing about COVID-19 and related factors.

Second, our sample can only represent the situation of Chinese users who shared verified information about COVID-19, which has certain limitations. Future research can consider validating our research model in other nations who have participated in sharing verified information about COVID-19 on SNS to reflect the differences between SNS users from different countries.

Third, our research model can only represent the situation of sharing verified information about COVID-19 on SNS. It cannot be applied in other research contexts directly. Therefore, it would be interesting for future studies to consider providing a general framework that not only can be applied to the COVID-19 context, but that can be used for other research contexts.

Finally, more factors could be considered to study the sharing of verified COVID-19 information. The previous literature has revealed many factors that have effects on sharing information or knowledge in other contexts which could be tested in our study. Therefore, future research can expand our findings, including factors such as rewards, reciprocity, and online social support since the effects of these factor have been demonstrated in information sharing literature in other contexts [15,37].

## 7. Conclusions

Sharing verified information about COVID-19 on SNS is important for both SNS users and the whole SNS to fight against the negative impacts of the COVID-19 pandemic. This study explores factors affecting users’ sharing of verified information about COVID-19 on SNS based on social exchange theory. From both the benefits and costs perspectives, we establish a research model to understand users’ sharing of verified information about COVID-19. Through the use of a survey method, we validate our proposed research model. The analysis results convey that to encourage the sharing of verified information about COVID-19 on SNS, we should consider both benefit and cost factors, not just factors from one perspective. SNS can use gamification elements in their design to increase user enjoyment and facilitate the sharing of verified information, and create a convenient, fast and reliable sharing environment to effectively reduce verification and executional costs.

## Figures and Tables

**Table 1:** Demographic information of sample participants.

Characteristics	Number	Percentage
Age		
<25	75	21.6%
25–30	88	25.4%
>30	184	53.0%
Gender		
Male	169	48.7%
Female	178	51.3%
Education		
High school	10	2.9%
College	304	87.6%
Master degree and above	33	9.5%
Length of use of social network sites during a day		
<2 h/day	86	24.8%
2–4 h/day	197	56.8%
>4 h/day	64	18.4%
Experience using social network sites		
<1 year	4	1.2%
1–5 years	88	25.3%
More than 5 years	255	73.5%

**Table 2:** Construct reliability and convergent validity.

Construct	Items	Factor Loadings	Composite Reliability	Average Variance Extracted	Cronbach’s Alphas
Enjoyment in helping (EH)					
	EH1	0.794	0.834	0.627	0.702
	EH2	0.782			
	EH3	0.799			
Reputation (RN)					
	RN1	0.723	0.876	0.640	0.814
	RN2	0.817			
	RN3	0.821			
	RN4	0.834			
Altruism (AM)					
	AM1	0.901	0.869	0.768	0.701
	AM2	0.851			
Verification cost (VC)					
	VC1	0.924	0.907	0.830	0.796
	VC2	0.898			
Executional cost (EC)					
	EC1	0.921	0.870	0.770	0.709
	EC2	0.831			
Verified information sharing (VIS)					
	VIS1	0.784	0.839	0.635	0.713
	VIS2	0.814			
	VIS3	0.792			

**Table 3:** Discriminant validity.

	AM ^a^	EC ^b^	EH ^c^	RN ^d^	VC ^e^	VIS ^f^
AM	0.877 ^g^					
EC	−0.187	0.877 ^g^				
EH	0.436	−0.282	0.792 ^g^			
RN	0.356	−0.164	0.483	0.800 ^g^		
VC	−0.089	0.298	−0.156	−0.143	0.911 ^g^	
VIS	0.391	−0.308	0.561	0.454	−0.213	0.797 ^g^

^a^ AM: Altruism, ^b^ EC Executional cost, ^c^ EH: Enjoyment in helping,^d^ RN: Reputation, ^e^ VC: Verification cost, ^f^ VIS: Verified information sharing, ^g^ The square roots of average variances extracted.

**Table 4:** Results of Hypotheses Testing.

Hypotheses	Path Coefficient	T-Statistics	*p*-Value	Supported
EH→SVI	0.357	6.408	0.000	Yes ***
AM→SVI	0.133	2.277	0.029	Yes *
RN→SVI	0.202	3.497	0.000	Yes ***
VC→SVI	−0.078	2.004	0.046	Yes *
EC→SVI	−0.126	2.534	0.011	Yes *

Notes: * *p* < 0.05, *** *p* < 0.001.

## Data Availability

The data presented in this study are not publicly available due to privacy and ethical.

## Supplementary Materials

The following are available online at https://www.mdpi.com/1660-4601/18/3/1260/s1, Table S1: Measurement instrument

## Appendix A

**Table A1:** Literature review.

Categories	Authors	Description	Conclusion
sharing information on COVID-19 in SNS	Verma [3]	A study of disinformation and misinformation sharing on COVID-19 exists in SNS.	sharing verified information on COVID-19 is not studied well and need further exploration.
	Yagnik [17]	A study of disinformation and misinformation sharing on COVID-19 exists in SNS, which can cause panic and anxiety for the panic.	
	Islam et al. [18]	A study of entertainment and self-promotion could promote the sharing of misinformation on COVID-19.	
	Apuke and Omar [19]	A study of altruism, instant news sharing, socialisation predicted fake news sharing related to COVID-19 pandemic among social media users in Nigeria.	
	Laato et al. [6]	A study of a person’s trust in online information and perceived information overload were strong predictors of unverified information sharing.	
information sharing in other contexts	Park et al. [21]	A study of two types of user behavior – information seeking and information sharing in online investment communities.	
	Baihaqi and Sohal [20]	A study of assessing several factors that influence the degree of information sharing in supply chains, namely integrated information technologies, internal integration, information quality and costs–benefits sharing.	
	Oh and Syn [23]	A study of enjoyment, self-efficacy, altruism, community interest, reciprocity and reputation could encourage users’ information sharing.	
	Lin et al. [24]	A study of social media design features, security and personal factors were the determinants that will affect users’ attitude and information sharing behavior.	
knowledge sharing.	Chang and chuang [25]	A study of combining the theories of social capital and individual motivation to investigate the factors influencing knowledge sharing behavior in a virtual community.	
	Yan et al. [15]	A study of members in online health communities shared knowledge from the perspective of benefits and costs.	
	Kankanhalli et al. [26]	A study of factors from benefit and cost perspectives significantly impacted EKR usage by knowledge contributors.	
