# Supplementary material for: Sharing of Verified Information about COVID-19 on Social Network Sites: A Social Exchange Theory Perspective
Source: Int J Environ Res Public Health. 2021 Jan 31;18(3):1260. doi: 10.3390/ijerph18031260 (PMC7908377; doi:10.3390/ijerph18031260)
Supplement: Supplementary file 1 [file ijerph-18-01260-s001.pdf]

Table S1. Measurement instrument.

Constructs	Items
Enjoyment in helping	• I enjoy sharing verified information about Covid-19 with others in SNS.<br>• It feels good to help someone else by sharing verified information about Covid-19 in SNS.<br>• Sharing verified information about Covid-19 with others in SNS gives me pleasure.
Reputation	• Sharing verified information about Covid-19 improves my reputation within in SNS.<br>• Sharing verified information about Covid-19 in SNS improves another people recognition of me.<br>• When I share verified information about Covid-19 in SNS, other people will respect me.<br>• When I share verified information about Covid-19 in SNS, other people will praise me.
Altruism	• I like to help other people in the SNS.<br>• I feel happy to support other people to solve Their Concerned problems in the SNS.
Verification cost	• The effort is high for me to verified information about Covid-19.<br>• Verified information about Covid-19 will cost me many resources (e.g., Network usage and related expertise).
Executional cost	• I can't seem to find the time to share verified information about Covid-19 in the SNS.<br>• It is laborious to share verified information about Covid-19 in the SNS.
Verified information sharing	• I intend to verify information of COVID-19 on SNS in the future.<br>• I plan to share verified information of COVID-19 on SNS.<br>• I will try to share verified information of COVID-19 on SNS in my daily life.
